# Telemedicine and Digital Health Applications in Vascular Surgery

**DOI:** 10.3390/jcm11206047

**Published:** 2022-10-13

**Authors:** Fabien Lareyre, Hava Chaptoukaev, Sharon C. Kiang, Arindam Chaudhuri, Christian-Alexander Behrendt, Maria A. Zuluaga, Juliette Raffort

**Affiliations:** 1 Department of Vascular Surgery, Hospital of Antibes Juan-les-Pins, 06600 Antibes, France; 2 Inserm U1065, C3M, Université Côte d’Azur, 06200 Nice, France; 3 Data Science Department, EURECOM, 06410 Biot, France; 4 Bodyo, 06200 Nice, France; 5 Department of Surgery, Division of Vascular Surgery, Loma Linda University School of Medicine, Loma Linda, CA 92350, USA; 6 Bedfordshire-Milton Keynes Vascular Centre, Bedfordshire Hospitals NHS Foundation Trust, Bedford MK42 9DJ, UK; 7 Brandenburg Medical School Theodor-Fontane, 16816 Neuruppin, Germany; 8 Department of Vascular and Endovascular Surgery, Asklepios Clinic Wandsbek, Asklepios Medical School, 20043 Hamburg, Germany; 9 Institute 3IA Côte d’Azur, Université Côte d’Azur, 06204 Nice, France; 10 Clinical Chemistry Laboratory, University Hospital of Nice, 06001 Nice, France

**Keywords:** telemedicine, digital health, telehealth, e-health, m-health, vascular surgery, vascular disease, aortic disease, peripheral artery disease, carotid stenosis

## Abstract

Background: Telemedicine has the potential to revolutionize healthcare. While the development of digital health technologies for the management of patients with cardiovascular diseases has been increasingly reported, applications in vascular surgery have been far less specifically investigated. The aim of this review is to summarize applications related to telemedicine in vascular surgery, highlighting expected benefits, current limits and future directions. Methods: The MEDLINE database was searched using a combination of keywords to identify studies related to telehealth/telemedicine in three main pathologies, including aortic, peripheral artery and carotid disease. A comprehensive literature review was performed to identify the type of digital application, intended use, expected benefits, strengths and limitations. Results: Telemedicine can improve the management of patients through digital platforms allowing teleconsultation, telemonitoring or telecoaching. Intended use involved remote consultation with a vascular surgeon, applications to enhance education, self-management, follow-up or adherence to treatment or lifestyle changes. Conclusion: Telemedicine offers innovative perspectives to improve access to care in distant locations and optimize care through patients’ empowerment and personalized follow-up, contributing to the development of precision medicine. Huge efforts remain necessary for its implementation in daily clinical practice and involve ethical, legal, technical, economic and cultural considerations.

## 1. Introduction

Telemedicine, also known as telehealth, is an innovation that has attracted increasing interest over the past few years, with the potential to revolutionize the delivery of healthcare through the use of digital platforms [1,2]. It corresponds to the use of digital communication technologies to provide healthcare remotely [1,2] that may change and provide new insights in the management of care in all medical and surgical specialties. Telemedicine groups various applications, including teleconsultation, telemonitoring, telesurveillance, telecoaching, telerehabilitation, communication and dissemination of information to the patients, through educative or supportive applications [3]. Telemedicine is part of the digital health or “e-health” era, which refers to health services delivered or enhanced through the internet or related information technologies [4]. The technical supports used to enable telemedicine can be diverse, including computers, tablets and wearable devices such as smart watches, headsets, body sensors, trackers or mobile phones (also referred as “m-health” for mobile phone technology) [5]. Telemedicine offers health services provided remotely and patients can have direct access to them using their own device, which can increase efficiency for healthcare needs, while improving patient satisfaction [6]. Another avenue of remote technology is health kiosks, which are publicly accessible devices that can be integrated into clinical facilities or pharmacies. To increase accessibility, health kiosks can also be placed in community locations which allow “opportunistic” uses [6]. 

Vascular diseases are associated with atherosclerosis, multiple comorbidities and risk factors such as higher age, smoking status, arterial hypertension, dyslipidemia and diabetes [7,8,9,10]. Depending on the symptoms and severity of the disease, it may require surgical interventions to treat it. Patients with vascular diseases could benefit from telemedicine applications in different settings, including remote diagnosis, education, clarification to medical treatment and post-operative follow-up, all of which can increase compliance to overall healthier life-style habits and early detection of adverse events. The deployment of digital health technologies for the management of patients with cardiovascular diseases has been extensively reported [11,12,13,14,15]. Technological advances with applications derived from artificial intelligence have also attracted increasing interest in vascular surgery [16,17,18,19,20,21,22,23]. Despite being an active clinical area, analysis of current knowledge on the use of telemedicine in vascular surgery is paradoxically lacking. Studies published so far have presented technical innovations and applications of e-health and telemedicine in the main vascular diseases encountered in vascular surgery, including aortic disease, lower extremity artery disease (LEAD) and carotid disease. However, state-of-the-art analyzing fields of applications, expected impact for practice, as well as current limitations, have been so far scarcely investigated. 

The aim of this narrative review is to summarize academic publications reporting the use of telemedicine in vascular surgery, highlighting expected benefits and discussing current limitations. By focusing on the three main pathologies most frequently treated in vascular surgery departments (aortic disease, LEAD and carotid disease), we provide a state of the art and highlight future directions in the field.

## 2. Methods

**Figure 1:**
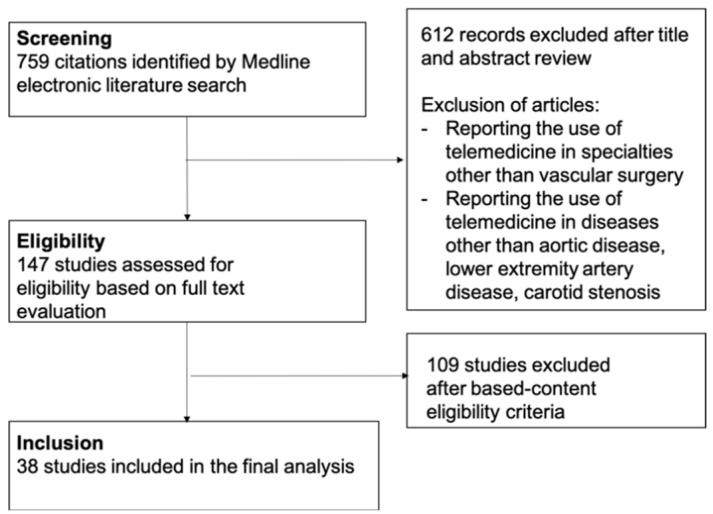
Flow chart depicting the process for the literature search and selection of the studies.

The MEDLINE database was independently searched by two authors (JR, FL) to identify studies reporting applications of telemedicine in vascular surgery and specifically focusing on three main vascular pathologies: aortic disease, LEAD and carotid stenosis. A combination of keywords was used, including “telehealth”, “telemedicine”, “vascular surgery”, “aortic”, “peripheral artery disease”, ‘lower extremity artery disease” and “carotid”. Only original articles published in the English language were included. After titles were identified, the abstracts were checked and full texts were retrieved. Exclusion criteria were studies that specifically focused on the use of telemedicine in diseases other than carotid, aortic or LEAD, or that reported the use of telemedicine in specialties other than vascular surgery. Eligibility was independently checked by two authors (JR, FL). In case of disagreement, the article was discussed with other authors to reach a consensus. The selection flow chart is depicted in Figure 1.

## 3. Telemedicine in Vascular Surgery

Healthcare systems currently face an increasing demand toward specialist consultations and it is estimated that the demand will soon exceed the specialist workforce [24]. In addition, the novel coronavirus (COVID-19) has imposed a severe strain on healthcare systems worldwide [25]. The high pressure on healthcare systems associated with social restrictions and hygiene measures challenged the management of patients with vascular diseases and forced institutions and health professionals to re-evaluate methods to provide appropriate and timely care to patients [26,27,28,29,30,31]. Telemedicine has been proposed as an alternative solution for the management of cardiovascular diseases [11,12,13,14,15] by potentially facilitating remote care for patients in distant locations, reducing congestion of health delivery institutions. Like many areas of medicine, vascular surgery has been transformed by the COVID-19 pandemic, with the rise of digital technologies to deliver appropriate care, while minimizing the potential spread of COVID-19 to patients, providers and the public [32]. The COVID-19 pandemic has led to an acceleration of telemedicine, remote monitoring, as well as digital health technologies, in various settings in vascular surgery, including education, clinical decision-making aids, data management and 3D imaging technologies [32].

In this context, several studies reported the use of teleconsultation in vascular surgery. Castaneda et al. performed a retrospective review on 350 teleconsultations completed by a single-center vascular surgery department [33]. They organized an established written service agreement between the vascular surgery service and primary care providers that established the indications and work-up for referral to vascular surgery [33]. Following the referral, the vascular specialist determined whether the patient required an in-person visit or could be evaluated through teleconsultation. The authors evaluated the outcomes of patients, as well as their compliance, with recommendations following the teleconsultation. In a cohort composed of 123 (35%) patients admitted for LEAD, 93 (27%) for carotid stenosis and 57 (16%) for abdominal aortic aneurysm (AAA), only 5.4% of the initial teleconsultations required conversion to an in-person visit [33]. Compliance of the patients with recommendations provided by physicians varied from 30% to 61% for medication recommendation, 57% for imaging recommendation and 5.1% for exercise recommendation [33]. Five-year all-cause mortality, as well as adverse events following teleconsultation, were comparable with reported complications in the literature. Taken all together, the authors concluded that teleconsultation was safe and efficient for triaging and providing recommendations for patients with vascular diseases, although the compliance of patients could greatly vary and needs to be further addressed. Another study analyzed care delivered at six vascular surgery telemedicine clinics over a 22-month period during the COVID-19 pandemic [34]. In total, 94 patients had 144 telemedicine visits, with the most common referrals being LEAD (20.2%) and AAA (14.9%). Telemedicine visit recommendations were: no intervention (31.9%), medical management (43.6%) and surgical intervention (24.5%) [34]. This retrospective analysis showed that telemedicine provided safe and efficient care and alleviated the travel burden for patients, with an average travel distance saved of 104 miles per telemedicine visit [34].

Other investigators aimed to evaluate whether telemedicine was as effective as direct patient examination for the development of a care plan in patients with vascular diseases. They blindly compared the evaluation of patients by two vascular surgeons: the first surgeon performing an in-person patient examination, the second one evaluating the patient remotely using telemedicine [35]. After performing 64 vascular evaluations in 32 patients, the results showed that teleconsultation was as effective as on-site evaluation in these patients, but it required technological support, with an on-site assistant, as well as surgeons trained and experienced in using the technology [35]. While efficiency of the digital platform and appropriate technical support are mandatory for teleconsultation, the perception and acceptance by patients is another consideration to address for a successful implementation in clinical practice. Lin et al. aimed to collect and evaluate patients’ feedback after a virtual visit with a vascular surgeon [36]. In this study, 10% of the patients chose the teleconsultation over in-person consultation, representing 55 patients with 82 remote clinical encounters over a 10-month period [36]. Diagnoses included arterial diseases (aneurysm, carotid and occlusive disease), as well as venous disease (deep venous thrombosis and varicose vein), and the consultations included 15 cases (18.3%) of first visits, 30 (36.6%) post-operative visits and 37 (45.1%) follow-up visits [36]. All patients who participated in the web-based satisfaction questionnaire found their virtual encounter more convenient than in-person visits and believed that they were able to communicate clearly with the health professionals [36]. Overall, 91% of them stated that they would recommend a virtual physician to a friend or a colleague [36] and one of the 82 remote encounters resulted in the need of an in-person office visit. These results suggest the safety of such an approach and show that telemedicine may be well-perceived and accepted by patients with vascular diseases [36]. Nevertheless, it should be noted that patients who participated in this study and chose telemedicine over face-to-face consultation might be more prone to using digital technology and might not be representative of all patients with vascular diseases. 

In addition to teleconsultation, other investigators aimed to develop applications for the follow-up after vascular surgery. Gunter et al. developed a mobile health application to follow wound healing in patients who had vascular surgery and tested its use on 40 participants with different surgical site locations, including groin, abdomen, lower extremity and amputation stump [37]. The patients were trained to use the mobile health application, which allowed them to transmit digital images of their surgical wound and answer a survey about their recovery [37]. Healthcare providers reviewed submissions daily and contacted patients when required. In total, seven wound complications were detected with one false negative [37]. The satisfaction of participant and care providers were ranked as high, suggesting interest in the application for post-operative telemonitoring. Finally, several applications to enhance wound image characterization and diagnosis have been proposed under telemedicine [38,39,40,41]. Although further studies are needed, these applications could also potentially be of use for patients following vascular surgery.

Taken together, these results suggest that e-consultations could be an alternative solution to enable triage or follow-up of patients by increasing the efficiency of access to vascular surgeon specialists without compromising the quality of care. Patients with vascular diseases can be old, frail or functionally limited. Telemedicine could potentially be useful by reducing burden, time and cost for long-distance travelling in selected patients. In addition, it could facilitate access to specialized care in regions with a low density of healthcare structures and providers. Finally, such technological support could help to develop a way to identify and treat patients with low-complexity and low-acuity vascular diseases, contributing to the reduction of clinic congestion and burden on health institutions, potentially resulting in increased efficiency and reduced time to treatment of acute conditions [33].

While the above studies underline the potential benefits of telemedicine for clinical practice, several issues still need to be addressed. Further evidence generation is required and the validation of the applicability, safety and efficiency of teleconsultation should be tested in larger multicentric international cohorts of patients to address the generalizability of the method in diverse regions, populations and health institutions [42]. Guidelines and clear consensus on standardized methods to achieve it are needed. Regarding feasibility, appropriate technological user support is required and training may be necessary for an optimal use by patients and health professionals. Several studies have highlighted that telemedicine uptake is lower in elderly patients and can be influenced by social and economic factors [43]. Vascular patients are often old and multimorbid and may be less at ease or may not even possess digital tools. Therefore, particular attention should be paid to maintaining equity of access care and avoiding exacerbating disparities among disadvantaged groups of patients [32]. Health professionals may also need training for a safe use of the applications developed. Such an approach thus requires a consequent investment for professionals and health institutions to develop hybrid clinics offering both telemedicine and traditional care adapted to the patients’ needs. 

Another issue is medicolegal and raises several considerations. First, the digital system should guarantee data protection, respect of privacy and confidentiality of medical information in accordance with the General Data Protection Regulation (GDPR). The system must guarantee adequate security and safeguards and data processing agreements may be required for the use of telemedicine platforms [44]. In addition, the matter of medical responsibility and insurance coverage needs to be addressed and may differ from one country to another. Legal implications in the case of misdiagnoses, failure to identify acute conditions and emergencies and potential delay in the delivery of healthcare under the use of telemedicine are issues that should be carefully taken into consideration, with legal frameworks that still need to be clearly defined. Like all medical and surgical specialties [45,46,47], vascular surgeons should be cautious and systematically check if their medical liability insurance policy covers telemedicine services. 

The cost to implement such technology, as well as its reimbursement from insurers or government programs, needs to be evaluated and balanced with the individual and collective benefits in terms of public health. The indications should also be precisely defined, depending on the type of vascular disease, symptoms and stage, as well as the intended use (i.e., first visit, diagnosis, follow-up, post-operative visits). An accepted standardized approach has yet to be developed. Finally, culture change, adherence of patients and health professionals, as well as the potential impact on the doctor–patient relationship, need to be further investigated [48]. Some may fear that telemedicine may negatively impact on the quality of care, expose patients to risks of malpractice or alter professional liability and integrity [45]. Communication, education and transparency are therefore crucial to establish and maintain trust in the security of the applications developed and successfully move to their implementation in daily clinical practice. 

## 4. Telemedicine and Aortic Disease

### 4.1. Teleconsultation

The above studies pointed to the interest of teleconsultation and telemonitoring in vascular surgery and included patients with various vascular diseases, including AAA [33,35,36]. An additional study specifically reported the use of a telehealth program to remotely perform pre-operative evaluation and post-operative follow-up in patients who underwent aortic surgery [49]. Among the 109 patients who underwent surgery (39% for aorto-iliac occlusive disease and 61% for AAA), eight (7.3%) required re-admission at the 30-day follow-up [49]. Four complications could be managed locally and four patients (3.6%) required transfer back to the operative hospital for additional care. Patient compliance was high, with only five (4.6%) patients who were non-compliant or lost to follow-up [49], confirming the potential interest of telemedicine for remote aortic surveillance in the pre and post-operative setting. To ease the burden of travel, patients may obtain medical images (i.e., computed tomography angiography or magnetic resonance imaging) in their local imaging facility and have their images uploaded to a tertiary medical center for review by specialists. Telemedicine then allows the reviewing vascular surgeon to create a digital encounter with the patient. Telemedicine can allow for surgeons to continue to provide quality care to patients in remote areas, while easing the physical constraints of distance and time.

Multidisciplinary consultation allows surgeons to discuss complex cases [7,8] and teleconsultation can be an asset to favor interactions between specialists across institutions. Chisci et al. reported a one-year experience of a regional service model of teleconsultation for planning and treatment of complex thoracoabdominal aortic disease. In this study, the surgeons had access to CT scans and clinical details using a web platform and they had a telemeeting to reach a final agreement on the operative strategy. This study performed on 24 cases suggested the potential interest in telemeeting to standardize the treatment of complex thoracoabdominal aortic disease in a huge region under the same health provider [50]. It also pointed to the interest in telemedicine to enhance medical education and to allow health professionals to share and discuss best practices more easily [50]. Although it is not systematically reported in the literature, remote multidisciplinary meetings between health professionals from different institutions have become increasingly used.

### 4.2. Digital Tools for Information and Education of Patients with Aortic Disease

Digital health technology has brought new tools to share medical knowledge, with a growing interest in applications to empower patients and provide them with appropriate information and education on their disease [51,52]. With that aim, some authors developed an educational e-health tool for patients with AAA and assessed its content and usability through questionnaires and focus group interviews [53]. Healthcare professionals’ feedback indicated that the content corresponded to the information that they usually provide to patients. Users’ feedback showed that the understandability and content was adequate, suggesting that the tool could improve communication and access to information in these patients [53]. They further investigated the interest in this application to reduce anxiety in patients with AAA undergoing surgery [54]. A single-center randomized clinical trial involving 120 patients with AAA scheduled for surgical repair was performed and no effect on anxiety mean scores was observed in the intention-to-treat analysis (−1.21 vs. −0.54, *p* = 0.330), with only half of the patients involved in the intervention group using the provided e-health tool. In contrast, a decrease in anxiety mean scores was noted in those who used the app in the per protocol analysis (−2.00 vs. −0.54, *p* = 0.028) [54]. Although the e-health tool resulted in lower anxiety scores in the participants who used it, the adherence and uptake were low. The application users were younger and had a higher educational level [54]. These results suggest that further improvement of the content and interface is required for its adoption and usefulness for patients in real-life practice. Other investigators aimed to evaluate the psychological impact on daily living in patients undergoing surveillance to monitor AAA progression and identified the main factors affecting them positively and negatively [55]. Based on interviews, dialogues and workshops with patients, they developed an interactive mobile application to inform patients and support them moving positively regarding living with an AAA [55].

## 5. Telemedicine and Lower Extremity Artery Disease

### 5.1. Telemonitoring and Telecoaching to Enhance Exercise Program 

Supervised exercise therapy programs have been the standard treatment for LEAD but can be impaired due to lack of patients’ adherence or lack of access, as underlined during the COVID-19 pandemic [9,56]. Home-based exercise programs have been proposed as an efficient alternative option [56]. There is a growing interest to use telemedicine as an innovative tool to enhance both supervised and home-based exercise programs, with the development of wearable activity monitors and applications to enhance education and patients’ adherence [57,58,59,60,61]. Several reviews and systematic analyses provided an overview of randomized controlled clinical trials that aimed at investigating the efficiency of telemedicine-enhanced exercise programs on patients’ outcomes [57,58,59,60,61,62,63,64,65,66,67,68,69,70,71,72,73] (Table 1). 

Telemedicine applications used varied and included telemonitoring with wearable sensors and physical activity trackers [62,63,64,67,68], as well as telecoaching applications using smart technology (computers or mobile phones) [65,66,69,73]. The clinical trials included symptomatic patients with intermittent claudication; one study also involved patients who had previously received peripheral endovascular therapy [65] and one involved asymptomatic patients with LEAD [68]. The duration of the study and follow-up could vary from 6 weeks [73] to 12 months [67]. The main outcomes investigated were the changes in walking performances in the telemedicine intervention group compared to controls [62,63,64,65,66,67,68,69,73]. Several studies showed a significant improvement in walking distance in the intervention groups, as assessed by the mean or median walking distance [64,67] or 6 min walking distance [63,69,73]. Questionnaires to evaluate walking ability were also used. In their study, Mays et al. observed a significant improvement in the Walking Impairment Questionnaire (WIQ) scores in patients from the intervention group after 14 weeks compared to the control group [65]. In contrast, McDermott et al. did not find a significant difference between groups’ WIQ score, SF-36 physical functioning score or Patient-Reported Outcomes Measurement Information System (PROMIS) mobility questionnaire [72] after 9 months. The discordance between the two studies can be attributed to several factors. While they both used telemonitoring with wearable activity monitors and telecoaching, the devices and applications were different. The time of duration differed and the first study involved a smaller number of patients who had previously received peripheral endovascular therapy, or who presented with stable claudication. The results may thus apply only to this selected population for a short-term follow-up. 

Several clinical trials also demonstrated the efficiency of telemedicine-enhanced exercise therapy to improve claudication onset time and peak walking time compared to baseline in the intervention group [62,63,65,66]. Some studies highlighted that the improvement in claudication onset time was even greater in the intervention telemedicine group compared to controls [65,66]. Telemedicine-enhanced exercise programs also contributed to increased daily average cadence [62,63] or daily activity, as evidenced by increased steps per day or decreased daily sit/lie hours [68]. Nevertheless, some studies did not observe a significant difference in the daily step counts or minutes of activity per day between the intervention and control groups [66,73]. In addition to walking performances, the impact of telemedicine-enhanced exercise programs on patients’ quality of life was investigated and demonstrated a significant improvement in the intervention groups [64,67,69,73]. Finally, a few studies investigated the impact of telemedicine-enhanced exercise applications on cardiovascular metrics and demonstrated heterogeneous results. The impact on oxygen uptake assessed by the measurement of peak VO_2_ was investigated and some investigators observed a significant improvement in the intervention telemedicine group compared to controls [66], while other studies did not [62,65]. The discrepancy between the studies may be due to differences in exercise programs and time of follow-up. Another clinical trial involving 108 patients with symptomatic LEAD showed that only the telemedicine-enhanced home-exercise program demonstrated significantly improved the large artery elasticity index and time to minimum calf muscle saturation StO_2_ during exercise, while no significant improvement was observed with the standard supervised exercise program and the attention control group [63]. Other authors investigated the impact on endothelial reactivity and found a significant increase in the intervention group compared to baseline, whereas no change was observed before and after the program in the control group [68]. These results may suggest a positive effect of telemedicine-enhanced exercise programs on endothelial dysfunction. 

While the results of these studies suggest that telemedicine (including telemonitoring, telecoaching and educational programs) could help to enhance physical activity in patients with LEAD, the quality of evidence needs to be improved and the long-term effect should be further evaluated [59]. As an example, while studies involving a limited number of patients found a significant improvement in 6 min walking distance in the intervention groups compared to controls [63,69,73], no difference was found after 9 months in the multicenter randomized clinical trial published by McDermott et al. involving 182 patients [72]. These results could suggest that the positive effect of telemedicine-enhanced exercise applications on walking could be transient and not sustained over long-term periods. The compliance, interests and motivations of patients should also be further investigated. A survey involving 99 patients with LEAD investigated the barriers for physical activity, the needs of patients and their interests for the development of telemedicine-enabled exercise [74]. Among them, 93% owned a mobile phone, 76% had internet access and 67% stated they had interest in telecoaching to support exercise [74]. In another cross-sectional survey involving 326 patients treated for symptomatic LEAD, 67% of patients owned a smart phone, 43% used smartphone app and only 15% used m-health applications [75]. Only one out of five patients agreed that such technologies could help to improve their lifestyle [75]. Interest in and use of m-health applications were inversely correlated with age [74,75]. Hence, the lack of interest and/or the lack of digital literacy in the elderly population may be a serious limitation to their use and implementation in daily clinical practice. In addition, expectations regarding app content may differ between patients and health professionals [76]. A cross-sectional survey analyzing forms completed by 483 patients and 615 physical therapists found that the distribution of most preferred app components could differ between patients and physicians [76]. Manufacturers should therefore be cognizant of finding the right balance between patients’ expectations and motivations with clinical usefulness and interests. 

### 5.2. Telemonitoring and Telecoaching to Enhance Follow-up

Several studies have shown that telemedicine can improve control of cardiovascular risk factors [77,78,79] and telemonitoring could be of interest to follow and detect early complications in patients with LEAD. With that aim, some investigators developed a telehealth program and investigated its impact on cardiovascular outcomes in patients with LEAD [80]. In a retrospective cohort study of 391 patients with LEAD from a tertiary hospital in Taiwan, the authors found that the 1-year incidence of cardiovascular events, including acute coronary syndrome and stroke, was significantly lower in the telehealth program group compared to the control group [80]. In addition, the medical costs of the patients who underwent the telehealth program were not higher than the control group in terms of outpatient, emergency department, hospitalization or total costs [80]. Another telehealth program called “Control Telehealth Claudication Intermittent” (CONTECI) was developed for patients with LEAD in order to follow the manifestations of LEAD and provide recommendations depending on the responses to the questionnaires [81]. This clinical trial compared 75 patients who underwent the telehealth program based on patients’ self-management and 75 patients in the control arm who followed traditional in-person visits for the follow-up. Interestingly, the number of scheduled and emergency visits significantly decreased in the intervention group and complications were diagnosed more quickly [81]. Taken together, these studies suggest that patient education, by promoting their pro-activity and expertise, may help to better control and detect LEAD-related complications and associated cardiovascular events. 

Hence, telemonitoring and telecoaching bring new perspectives to the management of patients with LEAD by empowering the patients, allowing a personalized follow-up, improving compliance to lifestyle changes and enabling an early detection of associated complications. Nevertheless, integration of such technologies in patients with LEAD, known to frequently have comorbidities or be present in the elderly, needs to be carefully investigated. Indeed, it is possible that the published studies may have selection bias towards patients more motivated and more familiar with digital technology, thus lowering the scope for generalization of the results. In addition, despite the expected benefits of telemedicine, the cost-effectiveness needs to be further addressed and investigated in different countries and healthcare systems. Such an analysis should consider costs required to develop and implement the devices, balanced with effects in the healthcare system at the individual and collective level [82]. 

## 6. Telemedicine and Carotid Disease

The literature on the use of telemedicine for the management of carotid disease is still very poor. In addition to the studies previously cited that showed the interest of teleconsultation in vascular surgery and included patients with carotid stenosis [33,36], Robaldo et al. reported the use of telemedicine for the early post-operative follow-up in patients who underwent carotid endarterectomy [83]. Ninety patients who fulfilled the criteria were discharge one day after the surgery and were monitored at home using an electronic blood pressure meter and a web-based video conferencing every 4 h. They were compared to a control group of 498 patients discharged on the second day post-operation. No patients were re-admitted for major complications and the results of the study suggested the feasibility of using telemedicine for post-operative follow-up to reduce post-operative in-hospital length of stay [83]. Finally, the management of patients with carotid stenosis and decisions regarding carotid interventions often involve a multidisciplinary team that includes neurologists/stroke physicians, vascular surgeons and interventional radiologists [84]. Although it is not systematically reported in the literature, remote multidisciplinary discussions between specialists and the use of teleradiology services with picture archiving and communication systems (PACS) using image exchange protocols (IEP) have become common practice to optimize care of patients, especially in the context of stroke management [84,85,86,87,88]. 

The use of telemedicine specifically in patients with carotid stenosis is less abundant, which could be attributed to the fact that medical treatment mainly relies on cardiovascular risk management. A wide range of telemedicine applications have been developed for the follow-up and control of cardiovascular risk factors [11,13,14,15,77,78]. These tools can apply to a large population of patients with various atherosclerosis-related lesions, including coronary, carotid or peripheral artery disease. This could, at least partly, explain why only a few studies specifically developed telemedicine applications for patients with carotid stenosis, as they can already benefit from the various tools targeting cardiovascular risk factors in general.

## 7. Conclusions

Telemedicine has the potential to improve the management of patients in vascular surgery through digital platforms allowing teleconsultation, telemonitoring or telecoaching. Teleconsultation allows remote access to vascular surgeons to facilitate triage or follow-up of patients. Digital technology can also be used to facilitate remote multidisciplinary consultation between professionals across specialisms and institutions to optimize care. Telemonitoring and telecoaching applications can enhance patients’ education and self-management, contributing to improved compliance to medical treatment and lifestyle changes. While telemedicine applications developed in vascular surgery can potentially benefit and be applied to a wide range of vascular diseases, the intended use may need to be precisely defined depending on the disease and its severity and should be tailored to the patients’ needs. Expected benefits of telemedicine can indeed differ depending on the vascular disease explored. Studies published so far indicate that the intended uses of telemedicine were mainly consultation, follow-up and educational tools for aortic aneurysm; telemonitoring, surveillance and coaching for LEAD; and remote multidisciplinary care for carotid stenosis. 

This literature review highlighted the potential positive impact of telemedicine in vascular surgery. Expected benefits for patients include improvement of access to care in distant locations, reduction of time and distance burden, empowerment and personalized follow-up, contributing to the development of precision medicine. For professionals, potential beneficial impacts involve reducing congestion and burden on health institutions and personalized management, potentially resulting in the optimization and improvement of care. 

While digital health applications bring promising perspectives, there is still a long way to go for their full implementation in daily clinical practice. Further evidence generation is required to ensure their safety, efficiency and benefits and standardized methods to evaluate it are needed. In addition, guidelines are currently being built and a huge challenge remains to reach a consensus regarding appropriate methods to guarantee and evaluate data protection and safety, define medicolegal responsibilities and insurance coverage or re-imbursement. Adherence of patients and professionals to digital health technology remains a key point for a successful, safe and efficient use. Efforts should be oriented toward information, communication, training and education, keeping in mind that these applications should be reserved for selected patients depending on their health conditions and technical skills. Particular attention should be taken to maintain equity, especially for the vulnerable population of patients in vascular surgery departments, including the elderly, disabled or patients with socioeconomic disadvantages. Telemedicine should thus be considered as a complementary tool that can be implemented into health institutions in addition to traditional in-person visits to develop “hybrid clinics” to optimize and provide care tailored to the patients’ needs.

This study presents some limits and further perspectives can be suggested. The literature search focused on three main vascular diseases (aortic disease, LEAD and carotid stenosis) and only original articles written in English were included. Further analysis including other vascular diseases and looking for articles in other languages might be of interest to provide an overview of the use of telemedicine worldwide and investigate potential differences between countries. Telemedicine and digital health applications are not systematically reported through academic publications and further analysis of patents and start-up development might shed a complementary light on current trends in the field. Finally, remote training and teaching is also an emerging field that can be associated with telemedicine and recent studies have highlighted the interest in telementoring in vascular surgery [89,90,91]. Although further validation is required, that kind of innovation could contribute to the proposal of new strategies to support skill introduction and development of specialized care in remote medical facilities under the collaboration and supervision of reference centers. Altogether, telemedicine might bring new insights to vascular surgery, not only in the management of care, but also for the education and training of the future generation of surgeons.

## Figures and Tables

**Table 1:** Summary of clinical trials investigating the use of telemedicine-enhanced exercise programs for patients with lower extremity artery disease (LEAD).

Aim	Telemedicine Application	Methods	Main Outcomes Measured	Results	References
Compare changes in exercise performance and daily ambulatory activity in PAD patients with intermittent claudication after a home-based exercise program, a supervised exercise program and usual care control.	- Home-based exercise program with telemonitoring using a step activity monitor (StepWatch3TM, Cyma Inc., Mountlake Terrace, WA, USA) and an exercise logbook to record walking sessions.	- Study design: prospective, randomized, controlled clinical trial - Population: 119 patients randomized, 29 completed home-based exercise, 33 completed supervised exercise and 30 completed usual care control. - Duration: 12 weeks	- Claudication onset time and peak walking time obtained from a treadmill exercise test - Daily ambulatory cadences measured during a 7-day monitoring period.	- Both exercise programs increased claudication onset time and peak walking time, whereas only home-based exercise increased daily average cadence. - The changes in claudication onset time and peak walking time were similar between the two exercise groups. - The change in daily average cadence was greater with home-based exercise (*p* < 0.05).	Gardner 2011 [62]
Compare changes in exercise performance in patients with symptomatic PAD following a home-based exercise program, a supervised exercise program and an attention control group.	- Home-based exercise program with telemonitoring using a step activity monitor (StepWatch3TM, Cyma Inc., Mountlake Terrace, WA, USA) and an exercise logbook to record walking sessions.	- Study design: prospective, randomized, controlled clinical trial - Population: 180 patients randomized, 53 completed home-based exercise, 52 completed supervised exercise and 51 completed usual care control. - Duration: 12 weeks	- Claudication onset time and peak walking time - Submaximal exercise performance, daily ambulatory activity, vascular function, inflammation and calf muscle hemoglobin oxygen saturation (StO_2_)	- Both the home-based exercise program and the supervised exercise program demonstrated a significant increase from baseline in claudication onset time, peak walking time, 6 min walking distance, daily average cadence and time to minimum calf StO_2_. - Only the home-based exercise program had improvements from baseline in large artery elasticity index (*p* < 0.05).	Gardner 2014 [63]
Investigate the impact of provision of daily feedback with an accelerometer, in addition to supervised exercise therapy, on walking distance.	- Supervised exercise program with feedback using a Personal Activity Monitor (PAM) accelerometer (PAM B.V., Doorwerth, The Netherlands)	- Study design: multicenter randomized clinical trial - Population: 304 patients with intermittent claudication, randomized to 83 exercise therapy in the form of “go home and walk” advice (WA), 93 supervised exercise therapy (SET) and 76 SET with feedback. - Duration: 12 months	- Change in absolute claudication distance. - Change in functional claudication distance and results on the Walking Impairment Questionnaire (WIQ) and Short-Form 36 (SF-36) Health Survey	- The median change in walking distance between 12 months and baseline in meters was 110 (0–300) in the WA group, 310 (145–995) in the SET group and 360 (173–697) in the SET with feedback group (*p* < 0.001 WA vs. SET). - WIQ scores and relevant domains of the SF-36 improved significantly in the SET groups.	Nicolai 2010 [64]
Determine the efficacy of a community-based walking exercise program with training, monitoring and coaching components to improve exercise performance and patient-reported outcomes in PAD patients.	- Telemonitoring with a piezoelectric activity monitor (OrthoCare Innovations, LLC, Oklahoma City, OK), a spring-levered hip pedometer (Model AE120XL, Accusplit, Inc., Livermore, CA, USA) and a provided walking exercise log - Telecoaching with educational print materials and a video, social and behavioral methods and environmental auditing of local walking areas for each patient.	- Study design: randomized, controlled trial - Population: 20 patients who previously received peripheral endovascular therapy or presented with stable claudication: 10 in the intervention group, 10 in the control group - Duration: 14 weeks	- Peak walking time (PWT) on a grading treadmill - Claudication onset time (COT) and patient-reported outcomes assessed via the Walking Impairment Questionnaire (WIQ).	- Intervention group patients did not significantly improve peak walking time when compared with the control group patients - Changes in claudication onset time and WIQ scores were greater for intervention patients compared with control patients.	Mays 2015 [65]
Determine the effects on functional capacity and physical activity patterns of a 12-week m-Health program in PAD patients with intermittent claudication.	- m-Health application consisting of daily exercise prescription and patient education using smartphones and physical activity trackers.	- Study design: randomized, controlled trial - Population: 20 patients randomized - Duration: 12 weeks	- Peak VO_2_ and claudication onset time - Changes in physical activity measured by steps per day and minutes of exercise per week.	- m-Health patients significantly increased peak VO_2_ from 15.2 ± 4.3 to 18.0 ± 4.8 mL/kg/min (*p* ≤ 0.05), while usual care did not change. - Comparison of these changes resulted in a significant difference between groups for peak VO_2_. - Claudication onset time significantly increased in m-Health (*p* ≤ 0.05), with a significant difference among the groups - Neither steps per day nor minutes of activity reached significant differences between groups.	Duscha 2018 [66]
Evaluate the effect of using wearable activity monitors (WAMs) with supervised exercise programs in patients with intermittent claudication	- Supervised exercise program with feedback using wearable activity monitors	- Study design: randomized, controlled trial - Population: 37 patients with intermittent claudication - Duration: 3, 6 and 12 months.	- Maximum walking distance, claudication distance and quality of life	- Patients in the WAM group showed significant improvement in maximum walking distance at 3 and 6 months, which was sustained at 12 months. - The WAM group increased claudication distance and VascuQol score - Significantly higher improvements in mean walking distance were seen in the WAM group compared with the control group at 6 months and 12 months.	Normahani 2018 [67]
Examine the effects of a 12-week in-home self-monitored physical activity in patients with asymptomatic PAD	- Home-based exercise program with telemonitoring using a monitor (activPal™) for postural and stepping parameters.	- Study design: randomized controlled trial - Population: 38 patients randomized to attention control (AC) or a physical activity sedentary reduction (PASR) group using an interactive online 3-month program focusing on increasing lifestyle physical activity and decreasing sedentary behaviors. - Duration: 3 months	- Changes in endothelial reactivity, arterial stiffness, sedentary behaviors and upright and stepping activities in individuals with asymptomatic PAD	- The PASR group significantly decreased daily sit/lie hours, increased sit-to-stand transitions per day and increased daily step counts - Endothelial reactivity significantly increased in the PASR group.	Laslovich 2020 [68]
Evaluate changes in exercise performance using a smartphone app (TrackPAD) to support supervised exercise training in patients with PAD	- m-Health application (TrackPAD) consisting of exercise prescription, recording of patients’ feedback and achievements	- Study design: single-blinded, randomized controlled trial - Population: 39 patients with symptomatic PAD (Fontaine stage IIa/b), randomized into 19 in the intervention group and 20 in the control group - Duration: 3 months	- Changes in the 6 min walking distance - Changes in physical activity and quality of life.	- The intervention group increased their mean 6 min walking distance, while the control group decreased their mean distance after 3 months of follow-up. - The quality of life increased significantly in terms of “symptom perception” and “limitations in physical functioning.” - Users’ feedback showed increased motivation and a changed attitude toward performing supervised exercise training.	Paldan 2021 [69]
Determine whether a home-based exercise intervention consisting of a wearable activity monitor and telephone coaching improves walking performances	- Home-based exercise program with telemonitoring using wearable activity monitor - Telephone coaching	- Study design: randomized clinical trial - Population: 200 patients with PAD randomized: 99 in the intervention group, 101 in the usual control group - Duration: 9 months	- Change in 6 min walking distance, change in subcomponents of the Walking Impairment Questionnaire (WIQ), SF-36 physical functioning score, Patient-Reported Outcomes Measurement Information System (PROMIS) mobility questionnaire, objectively measured physical activity.	- No significant difference on the mean change from baseline to 9-month follow-up in the 6 min walk distance in the intervention group vs. the usual care group - No significant differences between groups in the WIQ score, the SF-36 physical functioning score, the PROMIS mobility or satisfaction with social roles scores.	McDermott 2018 [72]
Develop and pilot a group education program for promoting walking in people with intermittent claudication.	- Education program with a 3 h group-based education workshop and follow-up telephone support	- Study design: randomized clinical trial - Population: 23 patients with PAD (Rutherford category 1–3) - Duration: 6 weeks	- Daily steps (tri-axial accelerometer), walking capacity (six-minute walk test and Gardner treadmill test), and quality of life (Intermittent Claudication Questionnaire [ICQ]), interviews to assess the acceptability and usefulness of the program	- Compared with controls, the intervention group had superior walking capacity and quality of life at six weeks. - The daily step count did not differ between groups.	Tew 2015 [73]

## Abbreviations

AC	attention control
LAEI	large artery elasticity index
NS	non-significant
PAD	peripheral artery disease
PASR	physical activity sedentary reduction
SET	supervised exercise therapy
SF-36	Short-Form 36 Questionnaire
SUS	System Usability Scale
VascuQol	Vascular quality of life questionnaire
WIQ	Walking Impairment Questionnaire
WA	“go home and walk” advice group
WAM	wearable activity monitors

## References

[1] (2021). Telehealth is here to stay. Nat. Med..

[2] Dorsey E.R., Topol E.J. (2016). State of Telehealth. N. Engl. J. Med..

[3] van der Meij E., Anema J.R., Otten R.H., Huirne J.A., Schaafsma F.G. (2016). The Effect of Perioperative E-Health Interventions on the Postoperative Course: A Systematic Review of Randomised and Non-Randomised Controlled Trials. PLoS ONE.

[4] Marwaha J.S., Landman A.B., Brat G.A., Dunn T., Gordon W.J. (2022). Deploying digital health tools within large, complex health systems: Key considerations for adoption and implementation. NPJ Digit. Med..

[5] Park Y.-T. (2016). Emerging New Era of Mobile Health Technologies. Healthc. Inform. Res..

[6] Maramba I.D., Jones R., Austin D., Edwards K., Meinert E., Chatterjee A. (2022). The Role of Health Kiosks: Scoping Review. JMIR Med. Inform..

[7] Chaikof E.L., Dalman R.L., Eskandari M.K., Jackson B.M., Lee W.A., Mansour M.A., Mastracci T.M., Mell M., Murad M.H., Nguyen L.L. (2018). The Society for Vascular Surgery practice guidelines on the care of patients with an abdominal aortic aneurysm. J. Vasc. Surg..

[8] Wanhainen A., Verzini F., Van Herzeele I., Allaire E., Bown M., Cohnert T., Dick F., van Herwaarden J., Karkos C., Koelemay M. (2019). Editor's Choice—European Society for Vascular Surgery (ESVS) 2019 Clinical Practice Guidelines on the Management of Abdominal Aorto-iliac Artery Aneurysms. Eur. J. Vasc. Endovasc. Surg..

[9] Aboyans V., Ricco J.B., Bartelink M.E.L., Bjorck M., Brodmann M., Cohnert T., Collet J.-P., Czerny M., De Carlo M., Debus S. (2018). Editor’s Choice—2017 ESC Guidelines on the Diagnosis and Treatment of Peripheral Arterial Diseases, in collaboration with the European Society for Vascular Surgery (ESVS). Eur. J. Vasc. Endovasc. Surg..

[10] AbuRahma A.F., Avgerinos E.D., Chang R.W., Darling R.C., Duncan A.A., Forbes T.L., Malas M.B., Murad M.H., Perler B.A., Powell R.J. (2022). Society for Vascular Surgery clinical practice guidelines for management of extracranial cerebrovascular disease. J. Vasc. Surg..

[11] Ding E.Y., Pathiravasan C.H., Schramm E., Borrelli B., Liu C., Trinquart L., Kornej J., Benjamin E.J., Murabito J.M., McManus D.D. (2021). Design, deployment, and usability of a mobile system for cardiovascular health monitoring within the electronic Framingham Heart Study. Cardiovasc. Digit. Health J..

[12] Yeung A.W.K., Kulnik S.T., Parvanov E.D., Fassl A., Eibensteiner F., Völkl-Kernstock S., Kletecka-Pulker M., Crutzen R., Gutenberg J., Höppchen I. (2022). Research on Digital Technology Use in Cardiology: Bibliometric Analysis. J. Med. Internet Res..

[13] Gandapur Y., Kianoush S., Kelli H.M., Misra S., Urrea B., Blaha M.J., Graham G., Marvel F.A., Martin S.S. (2016). The role of mHealth for improving medication adherence in patients with cardiovascular disease: A systematic review. Eur. Heart J. Qual. Care Clin. Outcomes.

[14] Palmer M.J., Machiyama K., Woodd S., Gubijev A., Barnard S., Russell S., Perel P., Free C. (2021). Mobile phone-based interventions for improving adherence to medication prescribed for the primary prevention of cardiovascular disease in adults. Cochrane Database Syst. Rev..

[15] Akinosun A.S., Polson R., Diaz-Skeete Y., De Kock J.H., Carragher L., Leslie S., Grindle M., Gorely T. (2021). Digital Technology Interventions for Risk Factor Modification in Patients with Cardiovascular Disease: Systematic Review and Meta-analysis. JMIR mHealth uHealth.

[16] Lareyre F., Behrendt C.-A., Chaudhuri A., Lee R., Carrier M., Adam C., Lê C.D., Raffort J. (2022). Applications of artificial intelligence for patients with peripheral artery disease. J. Vasc. Surg..

[17] Lareyre F., Behrendt C.-A., Chaudhuri A., Ayache N., Delingette H., Raffort J. (2022). Big Data and Artificial Intelligence in Vascular Surgery: Time for Multidisciplinary Cross-Border Collaboration. Angiology.

[18] Li B., Feridooni T., Cuen-Ojeda C., Kishibe T., de Mestral C., Mamdani M., Al-Omran M. (2022). Machine learning in vascular surgery: A systematic review and critical appraisal. NPJ Digit. Med..

[19] Lareyre F., Lê C.D., Ballaith A., Adam C., Carrier M., Amrani S., Caradu C., Raffort J. (2022). Applications of Artificial Intelligence in Non-cardiac Vascular Diseases: A Bibliographic Analysis. Angiology.

[20] Javidan A.P., Li A., Lee M.H., Forbes T.L., Naji F. (2022). A Systematic Review and Bibliometric Analysis of Applications of Artificial Intelligence and Machine Learning in Vascular Surgery. Ann. Vasc. Surg..

[21] Raffort J., Adam C., Carrier M., Ballaith A., Coscas R., Jean-Baptiste E., Hassen-Khodja R., Chakfé N., Lareyre F. (2020). Artificial intelligence in abdominal aortic aneurysm. J. Vasc. Surg..

[22] Tambyraja A.L. (2020). Artificial intelligence in vascular surgery: The next gold rush or busted flush?. J. Vasc. Surg..

[23] Raffort J., Adam C., Carrier M., Lareyre F. (2020). Fundamentals in Artificial Intelligence for Vascular Surgeons. Ann. Vasc. Surg..

[24] Association of American Medical Colleges (2018). The Complexities of Physician Supply and Demand: Projections from 2018 to 2033.

[25] Adams J.G., Walls R.M. (2020). Supporting the Health Care Workforce During the COVID-19 Global Epidemic. JAMA.

[26] Griffin C.L., Sharma V., Sarfati M.R., Smith B.K., Kraiss L.W., McKellar S.H., Koliopoulou A., Brooke B.S., Selzman C.H., Glotzbach J.P. (2020). Aortic disease in the time of COVID-19 and repercussions on patient care at an academic aortic center. J. Vasc. Surg..

[27] Sterpetti A.V. (2022). Telemedicine for screening and follow-up of abdominal aortic aneurysm. J. Vasc. Surg..

[28] Nishath T., Wright K., Burke C.R., Teng X., Cotter N., Yi J.A., Drudi L.M., Case M., David C.C., Fasano M. (2022). Implementation of telemedicine in the care of patients with aortic dissection. Semin. Vasc. Surg..

[29] Gonzalez Gomez A., Mendez Santos I., Monivas Palomero V., Calvo Iglesias F. (2021). Telemedicine for patients with valvular heart disease or aortic disease in the era of COVID-19. Rev. Esp. Cardiol..

[30] Hemingway J.F., Singh N., Starnes B.W. (2020). Emerging practice patterns in vascular surgery during the COVID-19 pandemic. J. Vasc. Surg..

[31] Fankhauser G.T. (2020). Delivering high-quality vascular care by telehealth during the COVID-19 pandemic. J. Vasc. Surg..

[32] Lin J.C., Welle N., Ding J., Chuen J. (2021). A look to the future: Pandemic-induced digital technologies in vascular surgery. Semin. Vasc. Surg..

[33] Castaneda P.R., Duffy B., Andraska E.A., Stevens J., Reschke K., Osborne N., Henke P.K. (2020). Outcomes and safety of electronic consult use in vascular surgery. J. Vasc. Surg..

[34] Chen A.J., Yeh S.L., Delfin D., Hoal G., Barron N., Riedinger T., Kashanijou N., Lieland J., Bickel K., O’Connell J.B. (2022). Telemedicine and Vascular Surgery: Expanding Access and Providing Care Through the COVID-19 Pandemic. Am. Surg..

[35] Endean E.D., Mallon L.I., Minion D.J., Kwolek C.J., Schwarcz T.H. (2001). Telemedicine in vascular surgery: Does it work?. Am. Surg..

[36] Lin J.C., Crutchfield J.M., Zurawski D.K., Stevens C. (2018). Implementation of a virtual vascular clinic with point-of-care ultrasound in an integrated health care system. J. Vasc. Surg..

[37] Gunter R.L., Fernandes-Taylor S., Rahman S., Awoyinka L., Bennett K.M., Weber S.M., Greenberg C.C., Kent C.K. (2018). Feasibility of an Image-Based Mobile Health Protocol for Postoperative Wound Monitoring. J. Am. Coll. Surg..

[38] Chakraborty C., Gupta B., Ghosh S.K. (2017). Chronic Wound Characterization using Bayesian Classifier under Telemedicine Framework. Int. J. e-Health Med. Commun..

[39] Schnalzer B., Huber S., Sumerauer I., Preininger M., Alcalde B., Mischak R. (2022). Evidence-Based Mobile Wound Application to Support Professionals in State-of-the-Art Chronic Wound Treatment. Stud. Health Technol. Inform..

[40] Kostovich C.T., Etingen B., Wirth M., Patrianakos J., Kartje R., Baharestani M., Weaver F.M. (2022). Outcomes of Telehealth for Wound Care: A Scoping Review. Adv. Ski. Wound Care.

[41] Gamus A., Keren E., Kaufman H., Brandin G., Peles D., Chodick G. (2021). Telemedicine versus face-to-face care for treatment of patients with lower extremity ulcers. J. Wound Care.

[42] Stern A.D., Brönneke J., Debatin J.F., Hagen J., Matthies H., Patel S., Clay I., Eskofier B., Herr A., Hoeller K. (2022). Advancing digital health applications: Priorities for innovation in real-world evidence generation. Lancet Digit. Health.

[43] Gordon N.P., Hornbrook M.C. (2016). Differences in Access to and Preferences for Using Patient Portals and Other eHealth Technologies Based on Race, Ethnicity, and Age: A Database and Survey Study of Seniors in a Large Health Plan. J. Med. Internet Res..

[44] Murphy D. Telemedicine and GDPR. https://challenge.ie/challengeblog/telemedicine-and-gdpr.

[45] Solimini R., Busardò F.P., Gibelli F., Sirignano A., Ricci G. (2021). Ethical and Legal Challenges of Telemedicine in the Era of the COVID-19 Pandemic. Medicina.

[46] Montemurro N. (2022). Telemedicine: Could it represent a new problem for spine surgeons to solve?. Glob. Spine J..

[47] Gardiner S., Hartzell T.L. (2012). Telemedicine and plastic surgery: A review of its applications, limitations and legal pitfalls. J. Plast. Reconstr. Aesthetic Surg..

[48] Onor M.L., Misan S. (2005). The Clinical Interview and the Doctor–Patient Relationship in Telemedicine. Telemed. J. e-Health.

[49] Kronenfeld J.P., Kang N., Kenel-Pierre S., Lopez A., Rey J., Fisher F., Karwowski J., Bornak A. (2022). Establishing and maintaining a remote vascular surgery aortic program: A single-center 5-year experience at the Veterans Affairs. J. Vasc. Surg..

[50] Chisci E., de Donato G., Fargion A., Ventoruzzo G., Parlani G., Setacci C., Ercolini L., Michelagnoli S. (2018). One-year experience of a regional service model of teleconsultation for planning and treatment of complex thoracoabdominal aortic disease. J. Vasc. Surg..

[51] Morley J., Floridi L. (2019). Enabling digital health companionship is better than empowerment. Lancet Digit. Health.

[52] Kuwabara A., Su S., Krauss J. (2020). Utilizing Digital Health Technologies for Patient Education in Lifestyle Medicine. Am. J. Lifestyle Med..

[53] Nilsson O., Hultgren R., Letterstal A. (2020). eHealth tool for patients with abdominal aortic aneurysm: Development and initial evaluation. Scand. J. Caring Sci..

[54] Nilsson O., Stenman M., Letterstål A., Hultgren R. (2021). A randomized clinical trial of an eHealth intervention on anxiety in patients undergoing abdominal aortic aneurysm surgery. Br. J. Surg..

[55] Mikkelsen R.B.L., Damsgaard B., Dahl M. (2022). Patients’ perspectives show us how to care for their needs when living with an abdominal aortic aneurysm: Development of an eHealth solution. J. Vasc. Nurs..

[56] Waddell A., Seed S., Broom D.R., McGregor G., Birkett S.T., Harwood A.E. (2021). Safety of home-based exercise for people with intermittent claudication: A systematic review. Vasc. Med..

[57] Haveman M.E., Kleiss S.F., Ma K.F., Vos C.G., Unlu C., Schuurmann R.C.L., Bokkers R.P.H., Hermens H.J., De Vries J.-P.P.M. (2019). Telemedicine in patients with peripheral arterial disease: Is it worth the effort?. Expert Rev. Med. Devices.

[58] Nugteren M.J., Catarinella F.S., Koning O.H.J., Hinnen J.-W. (2021). Mobile applications in peripheral arterial disease (PAD): A review and introduction of a new innovative telemonitoring application: JBZetje. Expert Rev. Med. Devices.

[59] Aronow W.S., Avanesova A.A., Frishman W.H., Shamliyan T.A. (2022). Inconsistent Benefits from Mobile Information Communication Technology in Adults with Peripheral Arterial Disease. Cardiol. Rev..

[60] Kim M., Kim C., Kim E., Choi M. (2021). Effectiveness of Mobile Health–Based Exercise Interventions for Patients with Peripheral Artery Disease: Systematic Review and Meta-Analysis. JMIR mHealth uHealth.

[61] Chan C., Sounderajah V., Normahani P., Acharya A., Markar S.R., Darzi A., Bicknell C., Riga C. (2021). Wearable Activity Monitors in Home Based Exercise Therapy for Patients with Intermittent Claudication: A Systematic Review. Eur. J. Vasc. Endovasc. Surg..

[62] Gardner A.W., Parker D.E., Montgomery P.S., Scott K.J., Blevins S.M. (2011). Efficacy of quantified home-based exercise and supervised exercise in patients with intermittent claudication: A randomized controlled trial. Circulation.

[63] Gardner A.W., Parker D.E., Montgomery P.S., Blevins S.M. (2014). Step-Monitored Home Exercise Improves Ambulation, Vascular Function, and Inflammation in Symptomatic Patients with Peripheral Artery Disease: A Randomized Controlled Trial. J. Am. Heart Assoc..

[64] Nicolaï S.P., Teijink J.A., Prins M.H., Exercise Therapy in Peripheral Arterial Disease Study Group (2010). Multicenter randomized clinical trial of supervised exercise therapy with or without feedback versus walking advice for intermittent claudication. J. Vasc. Surg..

[65] Mays R.J., Hiatt W.R., Casserly I.P., Rogers R.K., Main D.S., Kohrt W.M., Ho P.M., Regensteiner J.G. (2015). Community-based walking exercise for peripheral artery disease: An exploratory pilot study. Vasc. Med..

[66] Duscha B.D., Piner L.W., Patel M.P., Crawford L.E., Jones W.S., Patel M.R., Kraus W.E. (2018). Effects of a 12-Week mHealth Program on Functional Capacity and Physical Activity in Patients with Peripheral Artery Disease. Am. J. Cardiol..

[67] Normahani P., Kwasnicki R., Bicknell C., Allen L., Jenkins M.P., Gibbs R., Cheshire N., Darzi A., Riga C. (2018). Wearable Sensor Technology Efficacy in Peripheral Vascular Disease (wSTEP): A Randomized Controlled Trial. Ann. Surg..

[68] Laslovich S., Alvar B.A., Allison M., Rauh M.J. (2020). Effects of Lifestyle Physical Activity on Vascular Function in Asymptomatic Peripheral Arterial Disease. Med. Sci. Sports Exerc..

[69] Paldán K., Steinmetz M., Simanovski J., Rammos C., Ullrich G., Jánosi R.A., Moebus S., Rassaf T., Lortz J. (2021). Supervised Exercise Therapy Using Mobile Health Technology in Patients with Peripheral Arterial Disease: Pilot Randomized Controlled Trial. JMIR mHealth uHealth.

[70] Harzand A., Vakili A.A., Alrohaibani A., Abdelhamid S.M., Gordon N.F., Thiel J., Benarroch-Gampel J., Teodorescu V.J., Minton K., Wenger N.K. (2020). Rationale and design of a smartphone-enabled, home-based exercise program in patients with symptomatic peripheral arterial disease: The smart step randomized trial. Clin. Cardiol..

[71] Paldán K., Simanovski J., Ullrich G., Steinmetz M., Rammos C., Jánosi R.A., Moebus S., Rassaf T., Lortz J. (2019). Feasibility and Clinical Relevance of a Mobile Intervention Using TrackPAD to Support Supervised Exercise Therapy in Patients with Peripheral Arterial Disease: Study Protocol for a Randomized Controlled Pilot Trial. JMIR Res. Protoc..

[72] McDermott M.M., Spring B., Berger J.S., Treat-Jacobson D., Conte M.S., Creager M.A., Criqui M.H., Ferrucci L., Gornik H.L., Guralnik J.M. (2018). Effect of a Home-Based Exercise Intervention of Wearable Technology and Telephone Coaching on Walking Performance in Peripheral Artery Disease: The HONOR Randomized Clinical Trial. JAMA.

[73] Tew G.A., Humphreys L., Crank H., Hewitt C., Nawaz S., Al-Jundi W., Trender H., Michaels J., Gorely T. (2015). The development and pilot randomised controlled trial of a group education programme for promoting walking in people with intermittent claudication. Vasc. Med..

[74] Cornelis N., Buys R., Fourneau I., Dewit T., Cornelissen V. (2018). Exploring physical activity behaviour—Needs for and interest in a technology-delivered, home-based exercise programme among patients with intermittent claudication. Vasa.

[75] Alushi K., Hinterseher I., Peters F., Rother U., Bischoff M.S., Mylonas S., Grambow E., Gombert A., Busch A., Gray D. (2022). Distribution of Mobile Health Applications amongst Patients with Symptomatic Peripheral Arterial Disease in Germany: A Cross-Sectional Survey Study. J. Clin. Med..

[76] van den Houten M.M.L., Spruijt S., Fokkenrood H.J.P., Scheltinga M.R.M., Teijink J.A.W. (2018). User Preferences for Mobile Health Interventions: A Survey among Intermittent Claudication Patients and Their Physical Therapists. Ann. Vasc. Surg..

[77] Soliman A.M. (2020). Telemedicine in the Cardiovascular World: Ready for the Future?. Methodist Debakey Cardiovasc. J..

[78] Battineni G., Sagaro G.G., Chintalapudi N., Amenta F. (2021). The Benefits of Telemedicine in Personalized Prevention of Cardiovascular Diseases (CVD): A Systematic Review. J. Pers. Med..

[79] Vernooij J.W., Kaasjager H.A., Van Der Graaf Y., Wierdsma J., Grandjean H.M., Hovens M.M., De Wit G.A., Visseren F.L. (2012). Internet based vascular risk factor management for patients with clinically manifest vascular disease: Randomised controlled trial. BMJ.

[80] Lee J.-K., Hung C.-S., Huang C.-C., Chen Y.-H., Wu H.-W., Yu J.-Y., Ho Y.-L. (2021). The Costs and Cardiovascular Benefits in Patients with Peripheral Artery Disease from a Fourth-Generation Synchronous Telehealth Program: Retrospective Cohort Study. J. Med. Internet Res..

[81] Davins Riu M., Borras Perez X., Artigas Raventos V., Palomera Fanegas E., Serra Prat M., Alos Villacrosa J. (2018). Use of Telehealth as a New Model for Following Intermittent Claudication and Promoting Patient Expertise. Telemed. J. e-Health.

[82] Greving J.P., Kaasjager H.A.H., Vernooij J.W.P., Hovens M.M.C., Wierdsma J., Grandjean H.M.H., Van Der Graaf Y., de Wit G.A., Visseren F.L.J. (2015). Cost-effectiveness of a nurse-led internet-based vascular risk factor management programme: Economic evaluation alongside a randomised controlled clinical trial. BMJ Open.

[83] Robaldo A., Rousas N., Pane B., Spinella G., Palombo D. (2010). Telemedicine in vascular surgery: Clinical experience in a single centre. J. Telemed. Telecare.

[84] Naylor A.R., Ricco J.B., de Borst G.J., Debus S., de Haro J., Halliday A., Hamilton G., Kakisis J., Kakkos S., Lepidi S. (2018). Editor's Choice—Management of Atherosclerotic Carotid and Vertebral Artery Disease: 2017 Clinical Practice Guidelines of the European Society for Vascular Surgery (ESVS). Eur. J. Vasc. Endovasc. Surg..

[85] Feil K., Rémi J., Küpper C., Herzberg M., Dorn F., Kunz W.G., Reidler P., Levin J., Hüttemann K., Tiedt S. (2021). Inter-hospital transfer for mechanical thrombectomy within the supraregional stroke network NEVAS. J. Neurol..

[86] Klingner C., Tinschert P., Brodoehl S., Berrouschot J., Witte O.W., Günther A., Klingner C.M. (2020). The Effect of Endovascular Thrombectomy Studies on the Decision to Transfer Patients in a Telestroke Network. Telemed. e-Health.

[87] Troisi N., Cincotta M., Cardinali C., Battista D., Alberti A., Tramacere L., Michelagnoli S., Chisci E. (2021). Reallocation of Carotid Surgery Activity with the Support of Telemedicine in a COVID-Free Clinic during COVID-19 Pandemic. Eur. Neurol..

[88] English S.W., Barrett K.M., Freeman W.D., Demaerschalk B.M. (2022). Telemedicine-enabled ambulances and mobile stroke units for prehospital stroke management. J. Telemed. Telecare.

[89] Antoniou S.A., Antoniou G.A. (2017). Surgical Telementoring as a Means to Disseminate Vascular Expertise Around the World. J. Endovasc. Ther..

[90] Lareyre F., Chaudhuri A., Adam C., Carrier M., Mialhe C., Raffort J. (2021). Applications of Head-Mounted Displays and Smart Glasses in Vascular Surgery. Ann. Vasc. Surg..

[91] Porretta A.P., Alerci M., Wyttenbach R., Antonucci F., Cattaneo M., Bogen M., Toderi M., Guerra A., Sartori F., Di Valentino M. (2017). Long-term Outcomes of a Telementoring Program for Distant Teaching of Endovascular Aneurysm Repair. J. Endovasc. Ther..

